# The influence of spider news on online information-seeking

**DOI:** 10.1371/journal.pone.0308169

**Published:** 2024-10-23

**Authors:** André-Philippe Drapeau Picard, Catherine Scott, Angela Chuang, Stefano Mammola

**Affiliations:** 1 Insectarium de Montréal—Espace pour la vie, Montréal, Québec, Canada; 2 Department of Natural Resource Sciences, McGill University, Sainte-Anne-de-Bellevue, Québec, Canada; 3 Department of Biology, Memorial University of Newfoundland, St. John’s, Newfoundland and Labrador, Canada; 4 Department of Forestry and Environmental Conservation, Clemson University, Clemson, South Carolina, United States of America; 5 Molecular Ecology Group, Water Research Institute, National Research Council of Italy, Verbania Pallanza, Italy; 6 National Biodiversity Future Center, Palermo, Italy; 7 Finnish Museum of Natural History, University of Helsinki, Helsinki, Finland; William & Mary, UNITED STATES OF AMERICA

## Abstract

1. Fear of spiders is a widespread condition often disproportionate to the actual danger spiders pose to humans. Likely rooted in evolutionary history, fear of spiders might also have a cultural component. Recent studies have shown that a significant fraction of spider-related media reports are misleading and sensationalistic. Information-seeking behaviours serve as common coping mechanisms for our fears and anxieties, yet the link between spider-related news stories and such behaviors remains unexplored.
2. We hypothesize that media reports foster concern about spiders, resulting in an increased awareness of spiders and health issues associated with them. We extracted 1486 reports in English from a public database providing a content-analysis of spider-related online traditional media reports published between 2010–2020. We examined whether the volume of spider-related queries in Google Trends, Wikipedia, and iNaturalist increased in the week following the publication of each news story.
3. Sensationalistic news stories were associated with a small, significant increase in search volumes, compared to non-sensationalistic ones. The search volume for “brown recluse” (*Loxosceles reclusa*), which are potentially dangerous spiders, was higher after the publication date of news related to human-spider encounters. There was a significant positive relationship between the number of spider-related news stories published in a given month and the traffic on target spider-related Wikipedia pages, especially so for the page on brown recluse spiders.
4. Our results suggest that traditional media have a detectable impact on the behaviour of the general public towards spiders, supporting the hypothesis that the fear of spiders is perpetuated by culture. Additionally, our findings indicate that information-seeking behaviour is a common response to learn about spiders and potentially fact-check spurious claims found in sensationalised news. By recognizing the role of media in shaping attitudes towards spiders and acknowledging the benefits of accurate representation, we can lay the foundation for a more informed and harmonious relationship between humans and spiders.

## Introduction

Spiders occur in virtually every terrestrial ecosystem, including most human dwellings [1, 2]. Encounters with spiders are thus frequent, often eliciting intense reactions in humans. In fact, spiders are widely feared invertebrates [3–6] and arachnophobia is the most widespread nature-related phobia [7].

However, the fear of spiders is largely exaggerated and disconnected from the real danger spiders pose to humans. Only 248 out of the >50,000 described spider species can cause severe envenomation in humans, representing less than 0.5% of all spiders known to science [8]. In Canada and the United States, spider envenomations requiring treatment are rare events, although their actual prevalence is difficult to measure [9–12]. Since there is no reliable way to conclusively attribute physical signs and puncture marks alone to spiders, medical conditions resulting in skin lesions are sometimes misdiagnosed as spider bites by physicians [13, 14]. The fear of spiders is potentially more harmful to humans than spiders themselves, as it generates anxiety (potentially incurring high socio-economic costs [15] and can result in collateral damage such as injuries and material damage (e.g, unintentional house fires [16, 17]).

Fear of spiders is thought to be rooted in human evolutionary history. For example, studies have shown that infants react more rapidly and strongly to pictures of spiders than they do to pictures of other organisms [18–20], suggesting that fear of spiders is an ancestral trait that evolved to help avoid life-threatening situations. Further research investigated spider morphological traits associated with negative emotions. For instance, Landova et al. [6] have shown that robust, large-bodied spiders such as tarantulas (Theraphosidae), are more likely to be associated with fear and disgust.

However, the hypothesis of an evolutionary origin to the fear of spiders has drawn criticism. First, there is no evidence that spiders were a threat to survival to our ancestors. As stated above, the objective risk of medically relevant spider bites is very low in most regions of the world, including North Africa or the Middle East, where *Homo sapiens* originated. Second, the methodology used in some research supporting the evolutionary hypothesis, especially those that studied the reactions of infants, has been questioned (see for example Denzer [21]. Third, there are no extant spiders presenting the traits identified as most frightening and disgusting by Landova et al. [6] in the cradle of humanity. Frynta et al. [5] hypothesized that the fear of spiders is a by-product of the fear of scorpions, as the latter do represent a threat to humans and our relatives in North Africa and the Middle East.

Importantly, fear of spiders might also have a strong cultural component. Indeed, there are cross-cultural differences in the degree to which students rate their fear of spiders [3, 22]. Davey [23] argues that spiders served as scapegoats during the Middle Ages, as a response to the anxiety caused by unexplained epidemics. The association between spiders and illness would then have been perpetuated through culture among European descendants. Modern medicine now provides a scientific explanation to disease propagation, but the fear of spiders could well be perpetuated by the often misleading and sensationalistic coverage of spiders in traditional and social media [17, 24]. On social media, sensationalistic news stories are shared more frequently than non-sensationalistic ones [24].

Information-seeking behaviours are a common coping mechanism for our fears and anxieties [25]. In fact, most phobic individuals use the internet to seek information to appraise their condition [7, 26]. Popular resources to seek information on the internet include the Google search engine and the Wikipedia website, both of which are in the top ten most visited websites globally [27]. For instance, Singer et al. [28] showed that media coverage of a topic is a driver of Wikipedia use. Community science platforms can also be used to get an identification and information on an organism [29, 30]. All three resources can thus be used to research information on spiders. However, while it could be assumed that news stories about spiders trigger such information-seeking behaviour, this link remains unexplored.

We explored this potential link by asking if the publication of spider-related news stories translates into a change in the behaviour of the general public towards spiders. We hypothesize that media reports foster concern about spiders, resulting in an increased awareness of spiders and health issues associated with them, rightly or not. To test this hypothesis, we used data from Google Trends, iNaturalist, and Wikipedia to examine whether the volume of spider-related queries increased following the publication of spider-related news stories. We further hypothesize that this pattern would also be observed at the species level for news stories about a given species.

## Materials and methods

### Data collection

We extracted news story data and metadata from Mammola et al. [31]. This database consists of a curated global collection of 5348 unique spider-related news stories published online between 2010 and 2020 (from 81 countries in 40 languages). Specifically, for each published news story about a spider-human encounter, the database includes a subjective assessment of sensationalism (sensationalistic/overstated media reports often use emotional, exaggerated words or expressions) and information on the circulation of the news source, location and type of human-spider encounter, presence of photographs (of spiders or bites), presence and type of errors, whether different kinds of experts (medical professionals, pest controllers, or arachnologists) were consulted. From this database, we selected all news stories written in English, namely 1486 reports published in 13 English-speaking countries, and focused on a subset of the variables (see Table 1) as potential predictors of information-seeking behaviour.

**Table 1:** Variables used in the models, their descriptions, and rationale for their inclusion in the models. Variable names, descriptions and values are taken from Mammola et al. [32] unless when otherwise stated.

Variable	Description	Associated rationale
Search term	The terms searched in Google Trends. Levels: “spider”, “spider bite”, “brown recluse” and “black widow” for Google Trends; “spider”, “spider bite”, “brown recluse” and “Latrodectus” for Wikipedia. This variable was not included in the model for iNaturalist given that only the search term “Araneae” was used. See S1 Table in S1 Appendix for details.	Searches for “spider bite” may be associated with fear/concern, particularly when news stories report spider bites, whereas searches for “spider” may be more neutral. We also expect high motivation to search for “black widow” and “brown recluse” following news stories about these species in North America because they are widely feared and potentially harmful to humans.
Circulation	The circulation of the media that published the news story. Levels: “Regional”, “(Inter)national”. Note that, with respect to Mammola et al. [32], we merged the levels “International” and “National” to balance the factor levels.	We expect newspaper circulation to be related to the number of people who read each news story, which in turn may influence search volume following publication.
Event type	Did the human-spider encounter result in a bite or deadly bite? Levels: “bite”, “encounter”. Note that, with respect to Mammola et al. [32], we merged the levels “Bite” and “Deadly bite” to balance the factor levels.	News stories reporting “bites” may lead to more searches because of fear/concern.
Sensationalism	Is the media report sensationalistic/overstated? Levels: “yes”, “no”	Sensationalistic news stories may lead to more searches because of fact-checking behaviour or alarmism.
Error	Does the media contain errors about the biology of spiders? Levels: “yes”, “no”	News stories that contain errors may lead to more searches because of fact-checking behaviour.
Spider expert interviewed	Was an expert consulted/capable of identifying the spider involved (e.g, arachnologist, entomologist, taxonomist)? Levels: “yes”, “no”	Fact-checking behaviour may be influenced by the presence of quotes from experts in news stories. News stories that contain quotes from spider experts may appear more trustworthy than those that do not.
Other expert interviewed	Was any other ‘expert’ consulted in the news (e.g, medical doctor, pest controller)? Levels: “yes”, “no”	Fact-checking behaviour may be influenced by the presence of quotes from experts in news stories.
Figure	Does the media contain figures/photos? Levels: “yes”, “no”	Photos of spiders or spider bites may influence fact-checking behaviour or fear/concern.

We extracted response variable data from three different sources: Google Trends, iNaturalist, and Wikipedia. For each news story and data source, we extracted search volume data for a 14-day period spanning the 7 days preceding publication and the 7 days following publication, beginning on the date of publication. We chose this timespan as most views of online articles happen within 24 hours after their publication [32], and there might be a lag between reading an article about spiders and researching information about spiders.

First, we extracted data on Google search queries using Google Trends. We interpreted Google Trends as a proxy for shallow information-seeking by users–googling is often a quick way to inform oneself about a given topic. We focused on news stories and searches from Canada and the United States (n = 633). Google Trends does not provide the absolute number of search queries, but rather the relative frequency of a particular search term for a given time period ranging from 0 to 100, with 100 as the highest search query volume. Google Trends data have been used in previous studies as indicators of public engagement with nature and environment conservation [8, 33, 34]. We screened associated requests for each search term for the study region and period 2010–2020), as search volumes can be affected by associations with subjects that are irrelevant to the focus of the study [35]. We used refined search terms to exclude terms unrelated to actual spiders (e.g. Spider-Man) from the results (details in S1 Appendix.

Second, we extracted Wikipedia page views data using the pageviews tool [36]. We interpreted Wikipedia data as a proxy for more thorough information-seeking by users, e.g. from users willing to delve deeper into a given topic following a search in Google. We extracted data for the pages “Spider”, “Spider bite”, “Brown recluse spider”, and “*Latrodectus*”, the genus associated with all widow spider species and the page where users are redirected when they search “black widow”, respectively. Wikipedia page views data can be filtered by language, but not by country, hence we focused here on all 1486 news stories published in English and extracted data for the English versions of the Wikipedia pages. Given the large sample size for Wikipedia, we expected results for the Wikipedia model to be more robust.

Finally, we extracted iNaturalist observation data for the order Araneae, which includes all spiders. Arguably the most widely used community science platform, iNaturalist is a “*social network designed for sharing biodiversity observations and connecting people with nature*” [37]. As a crowdsourced tool for the identification of organisms that is generally used by people with a deep interest in nature [30, 38], we interpreted uploads to iNaturalist as a proxy for more scientifically oriented information-seeking by users. Through the platform’s export tool, we extracted data for all spiders for Canada and the United States [39]. Since iNaturalist is often used to seek taxonomic identifications of organisms encountered by users, we hypothesized that users might upload more photos of spiders in response to spider-related news articles. Since a number of different spider taxa are often confused with *Latrodectus* and *Loxosceles* spiders, and since we are interested in observer perception rather than spider identity *per se*, we extracted data from all of Araneae to fully capture this response. We later filtered and analyzed observations using their upload date, i.e. the date when they were uploaded to the platform, rather than observation date (the date the photograph was taken). Again, we focused on the 633 news stories from Canada and the United States.

### Statistical analyses

We conducted all analyses in R version 4.3.0 [40], following the general protocol by Zuur and Ieno [41] for regression analyses.

First, and independently for Google Trends, Wikipedia, and iNaturalist, we tested whether the queries volume connected with each news story differed seven days before and after its publication. To this end, for each combination of news story and search term within each data source, we fitted a generalized linear model testing whether the search volume changed in the seven days after publication (including the date of publication) compared to the seven days before. In fitting these multiple generalized linear models, we specified a binomial distribution (suitable for proportional data) for Google Trends data, a negative binomial distribution (suitable for overdispersed count data) for Wikipedia data, and a Poisson distribution (suitable for non-overdispersed count data) for iNaturalist data. This way, for each news story, data source, and associated search term, we obtained an estimate of how the search period affected the intercept of the model (hereafter “intercept change in search volume”), whereby positive estimates indicate news stories for which we observed a greater search volume after the publication, and negative values *vice versa*.

Next, we modeled how different news-level attributes (see Table 1) affected the intercept change in search volume of each news–namely, if some news-level attributes could explain why some news stories drive an increase or decrease in online searches following their publication. To this end, we fitted three linear mixed models for Google Trends, Wikipedia, and iNaturalist data. These models tested whether the following eight news-level factors had an effect on the intercept change for search volume: search term, circulation of the news, country, event type, sensationalism, spider expert interviewed, other expert interviewed, errors in reporting, and inclusion of spider pictures (see Table 1 for a description of each attribute and associated expectations). The mixed part of the models allowed us to account for data non-independence by including two random factors: i) the newspaper in which each news story was published, controlling for the expectation that news from the same newspapers may exhibit more similar attributes and elicit more similar behaviours in news consumers than expected by chance; and ii) a factor combining the months and year of publication of each news story (e.g, “03_2018”, “12_2011”) to address pseudoreplication resulting from multiple news stories published within the same period. Note that we ended up excluding the newspaper random factor in the iNaturalist model due to convergence problems. We checked the fit of each model by inspecting residuals and fitted values with the R package ‘performance’ version 0.11.0 [42].

Finally, we constructed two generalized linear models to examine whether the number of published news stories could explain the variation of Wikipedia page views and observations uploaded on iNaturalist for a given month. Note that this analysis was not possible for Google Trends data, given these are proportional data. For Wikipedia data, we modeled the relationship between the cumulative number of hits in a given month as a function of the number of news stories and the search term. For iNaturalist data, we excluded the factor search term for the model structure as only the search term “Araneae” was used in gathering data. In both models, we specified a negative binomial distribution due to significant overdispersion with Poisson models (Wikipedia: dispersion ratio = 10623.545, Pearson’s Chi^2^ = 2549650.744, p < 0.001; iNaturalist: dispersion ratio = 14208.207, Pearson’s Chi^2^ = 3409969.737, p < 0.001.

## Results

We extracted data and metadata from 1486 news stories published in English from Mammola et al. [32]. From that number, 105 originated from Canadian media, and 537 from United States media. Looking at specific taxa, 276 news stories were associated with brown recluse spiders (*Loxosceles reclusa*), 184 with black widows (*Latrodectus* spp.), and the remaining news stories with other spider species (Table A in S2 Appendix).

**Fig 1:**
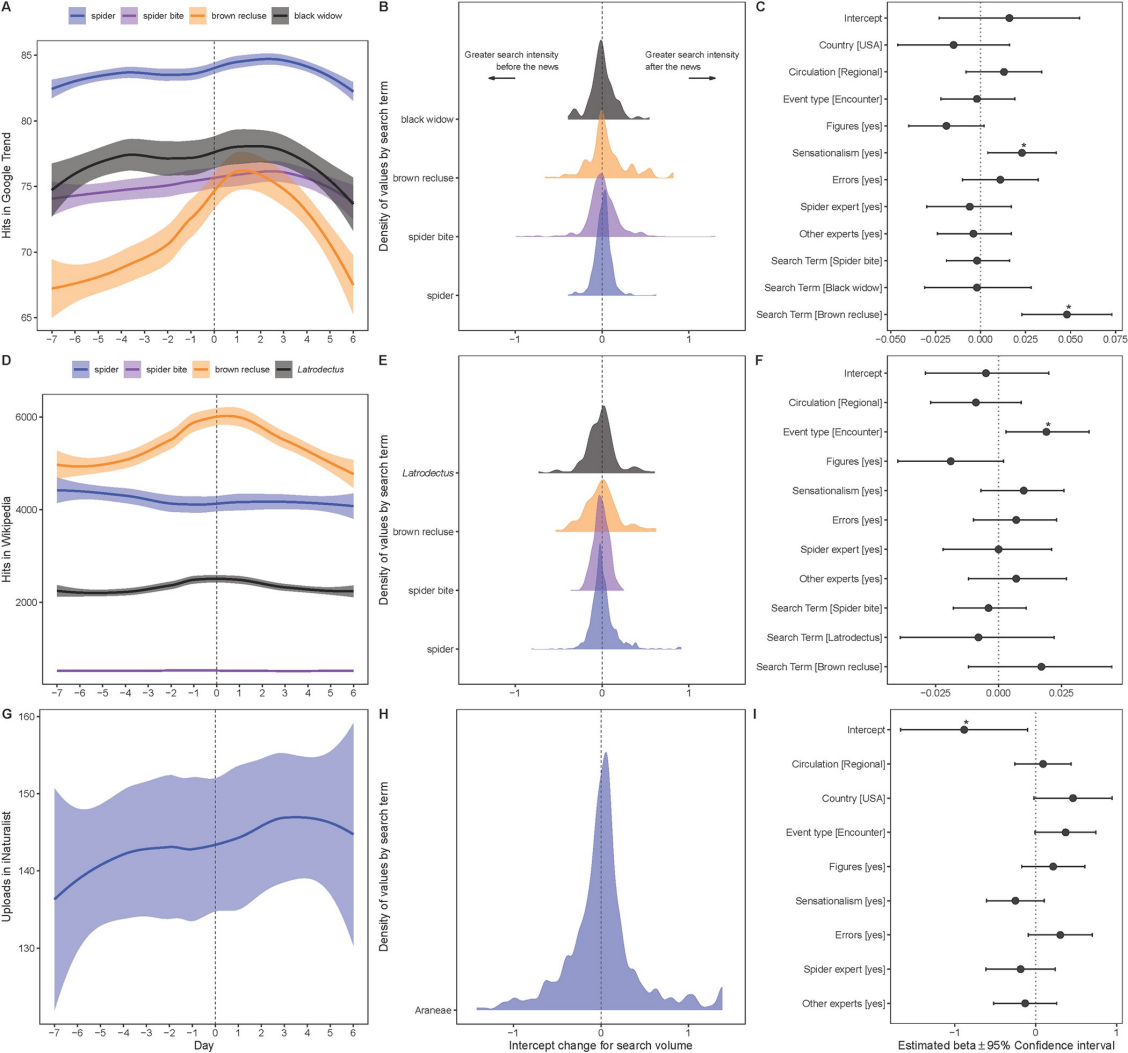
Results of analyses with Google Trends search volumes (A–C), Wikipedia page views (D–F), and iNaturalist observations (G–I). Left plots show daily search volume levels depicted by locally estimated scatterplot smoothing (LOESS) fits (filled lines) and 95% confidence envelopes (shaded surfaces), where day 0 corresponds to the date of news story publication. Center plots show density of values of change in search volumes after the publication date of each news. Right plots show estimated parameters (and 95% confidence interval) for linear mixed models testing the relationship between the intercept change in search volume after the publication of each news story and different news-level predictors. Exact model estimates are provided in Table B in S2 Appendix. * indicates p < 0.05.

We found a general pattern in Google Trends data, which showed an increase in search volume, although the relationship was not significant for all search terms (Fig 1A). Search volume for “brown recluse” was significantly higher during the week following the publication of news stories about that species compared to the baseline search term (spider) (Fig 1A and 1B). Sensationalistic news stories were associated with a significant increase in search volumes compared to non-sensationalistic ones (Fig 1C).

Wikipedia data showed that news stories involving a human-spider encounter were associated with an increase in page views following the publication of the stories compared to news stories involving biting events (Fig 1F). There were no significant differences in the views of the four pages studied. iNaturalist observation data showed no general change following the publication of news stories.

**Fig 2:**
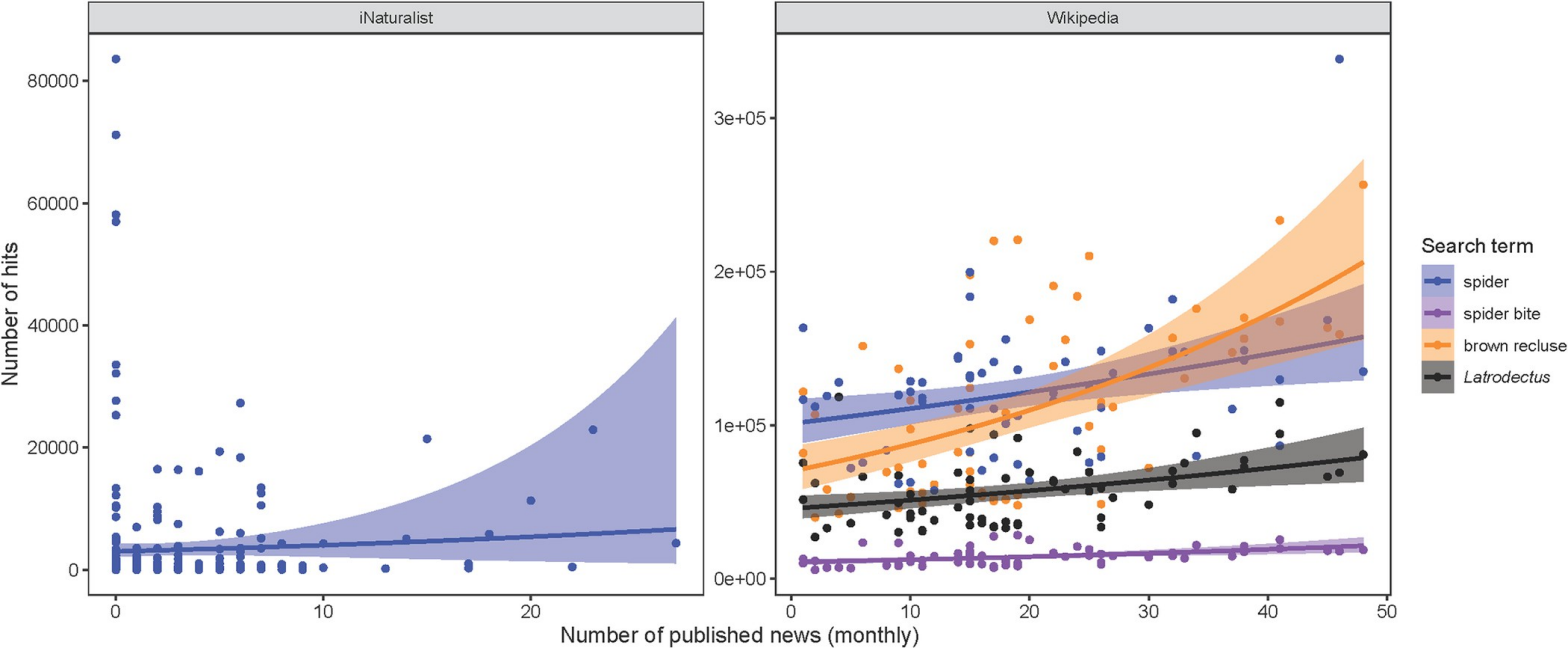
Negative binomial generalized linear models testing if the monthly number of news published has an effect on the number of spider (order Araneae) observation uploads on iNaturalist (left) and Wikipedia page views (right). Exact model estimates are given in Table C in S2 Appendix.

We found a positive relationship between the monthly number of news stories published and monthly Wikipedia page view numbers, but not spider observations on iNaturalist (Fig 2). The pattern was especially strong for the page “Brown recluse spider”.

## Discussion

### Interpretation of the observed patterns

The objective of our study was to determine whether a change in human behaviour could be detected following the publication of news stories reporting human-spider encounters. Our results support the hypothesis that some news stories have a measurable (although weak) effect on the online information-seeking behaviour of the general public. More broadly, our results tend to confirm that the fear of spiders has a cultural component.

Previous research has demonstrated that traditional media often depict spiders in a sensationalist and erroneous way [18, 25, 32, 43]. We provide evidence that media can also mediate human behaviour towards spiders, driving an information-seeking behaviour by the consumers of traditional news. It must be noted that we cannot tell whether increased online search activity by the news consumers after the publication of articles reflects simple curiosity, fact-checking of spurious claims, or a coping mechanism driven by arachnophobic sentiments (likely a combination of all these). Also, we acknowledge that most effect sizes in our models are rather weak, something that is expected but still begs some caution in the interpretation of our results.

Looking at Google search queries, we found that the search volume for “brown recluse” (i.e. *Loxosceles* spp.) was higher after the publication of most news stories related to human-spider encounters, but not for other search terms. Some spiders in the genus *Loxosceles* cause severe envenomations in humans [44]. As such, they often elicit strong arachnophobic sentiments and are depicted as highly dangerous in traditional and social media [25]–although hospitalisations are extremely rare [14]. Furthermore, sensationalistic news stories were associated with a significant increase in Google search volumes, compared to non-sensationalistic ones. Indeed, search engines are a known information source for people with anxiety [8, 27].

We found a strong relationship between the number of spider-related news stories published in a given month and the traffic on the spider-related Wikipedia pages we examined. This suggests that news stories have a cumulative effect on information-seeking behaviour. This could explain the weakness of the patterns observed when looking at the effect of single news stories, especially using noisy data. Taken together, our results show that media have a detectable impact on the behaviour of the general public towards spiders. This corroborates the findings by Singer et al. [29] that topics referenced in the media are often researched on Wikipedia.

### Towards a better representation of spider in the media

Two hypotheses have been formulated to explain why the fear of spiders is so widespread: the evolutionary hypothesis and the cultural hypothesis. Both are not mutually exclusive, but their respective contribution is unknown. While the evolutionary hypothesis has been questioned [22], evidence for the cultural hypothesis keeps accumulating. While these results do not inform us about the motivations or emotions behind the changes in information-seeking behaviour, they are in line with other findings in support for the cultural hypothesis, i.e. that attitudes towards spiders are modulated by the media.

If the media can be a driver of negative attitudes towards spiders and other widely feared animals (e.g, large carnivores [45, 46]), it can promote positive attitudes, too (e.g. as seen for bats [47]). Considering both the ecological importance of spiders (e.g. [48]) and the anxiety and collateral damage associated with arachnophobia [17, 49], the continued misrepresentation of arachnids is irresponsible. An effective way to achieve a better representation of spiders is through expert consultation by journalists when writing spider-related stories, which has been associated with a significant decrease in sensationalism [18]. Additionally, the use of emerging technologies such as large language models can be a costless and largely accessible way to fact-checking suspicious claims about spiders [50]. Moving away from sensationalism, traditional media have the power of fostering awareness about spider biology and contextualising perceived dangers, driving a positive transformation in societal perception and understanding of these too often misrepresented creatures.

Future studies could explore the impacts of media with different circulation levels at various spatial scales. It would be important to investigate whether the attitude of the readers is modulated by the tone of the articles themselves, i.e. positive, negative or neutral towards spiders. Other data sources, such as hashtag use on social media, could also be examined to see if similar patterns can be observed.

## Conclusion

We demonstrated that the portrayal of spiders in the media has the potential to affect information-seeking on spiders by news consumers. This holds implications for both public perception and ecological conservation. Only by recognizing the dual role of media in shaping attitudes towards these creatures and acknowledging the benefits of accurate representation can we lay the foundation for a more informed and harmonious relationship between humans and spiders. Through collaborative efforts between journalists, experts, and emerging technologies, we can foster a culture of understanding and appreciation for spiders, ultimately promoting their conservation [49].

## Supporting information

S1 Appendix Methodological details regarding data collection from Google Trends. (PDF)

S2 Appendix Supplementary results. Number of events (news story about a spider encounter) included in the analyses and estimated model parameters for the linear models.
(PDF)
