# Supplementary material for: The influence of spider news on online information-seeking
Source: PLoS One. 2024 Oct 23;19(10):e0308169. doi: 10.1371/journal.pone.0308169 (PMC11498699; doi:10.1371/journal.pone.0308169)
Supplement: S1 Appendix — (PDF) [file pone.0308169.s001.pdf]

The influence of spider news on online information-seeking (Supplementary material S1)

André-Philippe Drapeau Picard[1], Catherine Scott[2,3], Angela Chuang[4], Stefano Mammola[5,6]

[1]Insectarium de Montréal - Espace pour la vie, Montréal, Québec, Canada

[2]Department of Natural Resource Sciences, McGill University, Sainte-Anne-de-Bellevue, Québec

[3]Department of Biology, Memorial University of Newfoundland, St. John's, Newfoundland and Labrador, Canada

[4]Department of Forestry and Environmental Conservation, Clemson University, Clemson, South Carolina, United States of America

[5]Molecular Ecology Group, Water Research Institute, National Research Council of Italy (CNR), Verbania Pallanza, Italy

[6]National Biodiversity Future Center, Palermo 90133, Italy

[7]Finnish Museum of Natural History, University of Helsinki, Helsinki, Finland

ORCiD

APDP 0000-0003-0058-0124
AC 0000-0001-6847-5115
CS 0000-0003-0860-4805
SM 0000-0002-4471-9055

S1. Data collection from Google Trends

As words have multiple meanings, Google Trends search volumes can be affected by associations with subjects that are irrelevant to the focus of the study. It is therefore necessary to prune unrelated searches from the results when extracting data from Google Trends. To do so, we used the validation methodology proposed by Correia (2018). For each search term (spider, spider bite, brown recluse and black widow), for each country (Canada and USA) and for the whole period of the study (2010-2020), we first queried Google Trends using only the initial search term (Table S1). We then screened top related queries for topics unrelated to actual spiders. Those unrelated search terms were added to the query string to remove them from the results. For example, a query using only “spider” as the search term will include spider verse in the results but using “spider - verse” will exclude it. We ended up with refined search terms that we used to extract the data used in our analyses.

Table S1. Initial and refined search terms used for Google Trends data extraction.

Country	Initial search term	Refined search term
Canada	spider	spider - verse - spiderman - man - solitaire - minecraft - fiat - gwen - ferrari - tech - mclaren
Canada	spider bite	spider bite - verse - spiderman - man - solitaire - minecraft - fiat - gwen - ferrari - mclaren
Canada	brown recluse	brown recluse
Canada	black widow	black widow - avengers - marvel - iggy - superhero - razer
United States	spider	spider - man - solitaire - verse - minecraft - angolan - lucas - ferrari - gwen - romeo - fermented
United States	spider bite	spider bite - man - solitaire - verse - minecraft - angolan - lucas - ferrari - gwen - romeo
United States	brown recluse	brown recluse - brownells - browning
United States	black widow	black widow - iggy - avengers - razer - silverado - hulk - endgame - singer - captain - gmc - taskmaster

References

Correia RA. Google Trends data need validation: Comment on Durmuşoğlu (2017). Hum Ecol Risk Assess. 2018;25(3):787-90. doi: 10.1080/10807039.2018.1446322.
