# Supplementary material for: The influence of spider news on online information-seeking
Source: PLoS One. 2024 Oct 23;19(10):e0308169. doi: 10.1371/journal.pone.0308169 (PMC11498699; doi:10.1371/journal.pone.0308169)
Supplement: S2 Appendix — Number of events (news story about a spider encounter) included in the analyses and estimated model parameters for the linear models. (PDF) [file pone.0308169.s002.pdf]

The influence of spider news on online information-seeking (Supplementary material S2)

André-Philippe Drapeau Picard[1], Catherine Scott[2,3], Angela Chuang[4], Stefano Mammola[5,6]

[1]Insectarium de Montréal - Espace pour la vie, Montréal, Québec, Canada

[2]Department of Natural Resource Sciences, McGill University, Sainte-Anne-de-Bellevue, Québec

[3]Department of Biology, Memorial University of Newfoundland, St. John's, Newfoundland and Labrador, Canada

[4]Department of Forestry and Environmental Conservation, Clemson University, Clemson, South Carolina, United States of America

[5]Molecular Ecology Group, Water Research Institute, National Research Council of Italy (CNR), Verbania Pallanza, Italy

[6]National Biodiversity Future Center, Palermo 90133, Italy

[7]Finnish Museum of Natural History, University of Helsinki, Helsinki, Finland

ORCiD

APDP 0000-0003-0058-0124

AC 0000-0001-6847-5115

CS 0000-0003-0860-4805

SM 0000-0002-4471-9055

S2. Supplementary results

Table A. Number of events included in the analyses per data source country, search term. An event corresponds to a news story about a spider encounter, as defined and compiled by Mammola et al. (2022). Since Wikipedia page views data can be filtered by language, but not by country, we included all English news stories for this analysis. Note that Wikipedia page views data cannot be retrieved before July 2015, hence the shorter time range.

**Data source**	**Country**	**Search term**	**Time range**	**Number of events**
GTrends	Canada	Spider	2010-2020	96
GTrends	Canada	Spider bite	2010-2020	96
GTrends	Canada	Brown recluse	2010-2020	8
GTrends	Canada	Black widow	2010-2020	17
GTrends	United States	Spider	2010-2020	537
GTrends	United States	Spider bite	2010-2020	537
GTrends	United States	Brown recluse	2010-2020	218
GTrends	United States	Black widow	2010-2020	131
iNaturalist	Canada and United States	Araneae	2010-2020	627

Wikipedia	English-speaking countries	Spider	2015-2020	1207
Wikipedia	English-speaking countries	Spider bite	2015-2020	1207
Wikipedia	English-speaking countries	Brown recluse spider	2015-2020	209
Wikipedia	English-speaking countries	Latrodectus	2015-2020	177

Table B. Estimated model parameters for the linear model testing if different news-level factors have an effect on the intercept change for Google search volume, Wikipedia page views, and upload date of iNaturalist spider observations.

	Google Trends	Wikipedia	iNaturalist
Intercept	0.016 [-0.023, 0.055]	-0.005 [-0.029, 0.020]	-0.885 [-1.669, -0.101]
Circulation [Regional]	0.013 [-0.008, 0.034]	-0.009 [-0.027, 0.009]	0.090 [-0.257, 0.436]
Country [USA]	-0.015 [-0.046, 0.016]		0.461 [-0.023, 0.944]
Errors [yes]	0.011 [-0.010, 0.032]	0.007 [-0.010, 0.023]	0.304 [-0.092, 0.700]
Spider expert [yes]	-0.006 [-0.030, 0.017]	0.000 [-0.022, 0.021]	-0.187 [-0.617, 0.242]
Figures [yes]	-0.019 [-0.040, 0.002]	-0.019 [-0.040, 0.002]	0.216 [-0.173, 0.606]
Other experts [yes]	-0.004 [-0.024, 0.017]	0.007 [-0.012, 0.027]	-0.130 [-0.520, 0.260]
Sensationalism [yes]	0.023 [0.004, 0.042]	0.010 [-0.007, 0.026]	-0.251 [-0.607, 0.105]
Search Term [Brown recluse]	0.048 [0.023, 0.073]	0.017 [-0.012, 0.045]	
Search Term [Black widow]	-0.002 [-0.031, 0.028]		
Search Term [Latrodectus]		-0.008 [-0.039, 0.022]	

Search Term [Spider bite]	-0.002 [-0.019, 0.016]	-0.004 [-0.018, 0.011]	

Table C. Estimated model parameters for the linear model testing if the monthly number of news published has an effect on the number of spider observation uploads on iNaturalist and Wikipedia page views.

	iNaturalist	Wikipedia
Intercept	8.011 [7.693, 8.361]	11.426 [11.312, 11.541]
Number of news	0.029 [-0.027, 0.103]	0.014 [0.011, 0.018]
Search Term [Spider bite]		-2.146 [-2.273, -2.019]
Search Term [Brown recluse]		-0.106 [-0.234, 0.021]
Search Term [Latrodectus]		-0.754 [-0.882, -0.627]

Data availability statement

Data is available on Figshare (10.6084/m9.figshare.25810450). (LINK will become active upon acceptance)

R code to reproduce the analysis is available in GitHub (https://github.com/StefanoMammola/Drapeau-Picard-et-al-Spider-News-Trends) (LINK will become active upon acceptance)

References

Mammola, S., Malumbres-Olarte, J., Arabesky, V. *et al.* 2022b. An expert-curated global database of online newspaper articles on spiders and spider bites. *Sci Data* 9, 109. https://doi.org/10.1038/s41597-022-01197-6
